# Regulatory T cells for the control of physiological and pathological immune responses

**DOI:** 10.1186/1479-5876-9-S2-I16

**Published:** 2011-11-23

**Authors:** Shimon Sakaguchi

**Affiliations:** 1 Dept. of Experimental Pathology, Institute for Frontier Medical Sciences, Kyoto University, Kyoto, Japan

## 

Abstract not submitted for publication

